# Flavor Improvement of Maillard Reaction Intermediates Derived from Enzymatic Hydrolysates of *Oudemansiella raphanipes* Mushroom

**DOI:** 10.3390/foods13111688

**Published:** 2024-05-28

**Authors:** Furong Hou, Shasha Song, Wenjia Cui, Zipeng Yu, Zhiqing Gong, Yansheng Wang, Wenliang Wang

**Affiliations:** 1 Institute of Agro-Food Sciences and Technology, Shandong Academy of Agricultural Sciences, Jinan 250100, China; houfurong2010@163.com (F.H.); songshasha125@163.com (S.S.); clementpost@126.com (W.C.); gongzq413@tom.com (Z.G.); sdnky_wys@163.com (Y.W.); 2 College of Food Science and Engineering, Shandong Agricultural University, Taian 271018, China; 13176680080@163.com

**Keywords:** *Oudemansiella raphanipes*, Maillard intermediates, enzymatic hydrolysate, flavor substances

## Abstract

Maillard reaction intermediate (MRI) was prepared by the enzymatic hydrolysate (EH) of *Oudemansiella raphanipes* and fructose. The optimal preparation condition of MRIs was obtained when the Maillard reaction parameters were as follows: fructose addition of 5%, reaction time of 60 min, and temperature of 60 °C. E-Tongue results indicated that the umami and saltiness of MRIs were greater than those of Maillard reaction products (MRPs) and EH, and the taste-enhancing ability of MRIs was even more prominent than that of MRPs. E-Nose could obviously distinguish EH, MRIs, and MRPs, and there was an obvious difference between MRPs and MRIs regarding volatile aroma compounds. A total of 35 volatile flavor substances were identified among the three samples, including 6 alcohols, 13 aldehydes, 9 ketones, 2 esters, and 5 other compounds. Overall, MRIs could avoid the production of complete reaction products with an inferior flavor, and further enhance the umami taste.

## 1. Introduction

*Oudemansiella raphanipes*, commercially called “Changgengu” or “Heipijizong,” is a kind of well-known culinary edible mushroom in China with high economic value and excellent unique flavor [1,2]. Since its artificial cultivation in large quantities has been widely explored, *Oudemansiella raphanipes* is cultivated throughout China [3]. In addition to extensive research on germplasm resource, genetic diversity, cultivation, deep fermentation technology, and biological activity, numerous bioactive compounds produced by *Oudemansiella raphanipes* have been discovered, including polysaccharides, enzymes, orcinol, ergosterol, triterpenes, and other nutrients, manifesting its positive potential antioxidant, antitumor, immunomodulatory, and hepatoprotective role [4,5,6]. Recently, as its palatable taste and flavor compounds become increasingly well-known, *Oudemansiella raphanipes* is gaining a growing popularity. It is reported that the equivalent umami concentration of fresh *Oudemansiella raphanipes* is 4.72–23.66 monosodium glutamate/100 g [3], and appropriate processing and storage conditions may enhance the content of flavor substances. For example, it is found that cutting root treatment combined with low-temperature storage effectively maintains the content of phenolic, flavonoid, and C8 volatile substances of *Oudemansiella raphanipes*, obtaining high sensory scores and a high umami value and inhibiting the production of off-flavor acids [3].

The Maillard reaction is a non-enzymatic reaction that occurs between a carbonyl group (such as reducing sugar, aldehyde, and ketone) and an amino compound (such as amine, amino acid, peptide, and protein) at high temperatures [7], which can improve food flavor and stability [8,9]. Some researchers have found that the Maillard reaction can eliminate the bad flavor (such as foul smell, acetone-like odor, and aldehyde-like odor) of an edible mushroom’s enzymatic hydrolysate while improving the good flavor (such as mushroom smell, honey aroma, and fruity aroma) [8,10]. Additionally, Maillard reaction products (MRPs) always exert enormous influence on the flavor and quality of food, and hence, more and more studies are focusing on the application of MRPs to the preparation of flavors and condiments. However, the strong volatility of these compounds results in their instability during further thermal processing and the storage of food [11]. Generally, the Maillard reaction is divided into three stages. The initial stage starts with a condensation between an amino group and a reducing sugar, leading to the rearrangement of N-glycosylamine in the presence of the aldose sugar into the so-called Amadori compounds (or Heyns compounds when the reducing sugar is ketose). The intermediate stage starts from the Amadori/Heyns compounds and leads to the generation of sugar fragmentation products and the release of the amino groups. In the final stage of the reaction, many active substances will be produced, such as glucose ketones, reducing ketones, and acetone aldehydes, which could continue to react with amino compounds, ultimately producing brown polymer compounds, collectively called the dubbed melanoid [12]. Maillard reaction intermediates (MRIs), including Amadori or Heyns compounds, are extremely important nonvolatile aroma precursors with relatively stable chemical properties, but they tend to be degraded into flavor compounds at high temperatures [13]. A growing body of evidence suggests that MRIs would produce a fresh flavor during heat treatment as Maillard reaction proceeds [14]. Consequently, MRIs, as a desirable taste enhancer and flavor precursor, have a great prospect of application as substitutes of monosodium glutamate and MRPs. Furthermore, the preparation of MRIs could control flavor formation and browning during the Maillard reaction, which is highly preferred by consumers and pre-made food [15].

In this research, we prepared MRIs with reducing sugars and the enzymatic hydrolysate (EH) of *Oudemansiella raphanipes* and optimized the preparation process using the response surface methodology (RSM), aiming to provide a new method for improving the flavor of *Oudemansiella raphanipes*. The flavor characteristics of EH, MRIs, and MRPs were analyzed based on E-Tongue, E-Nose, and GC-IMS techniques, in the light of the absorbance, volatile and non-volatile flavor substances, and sensory evaluation. The results will be conducive to understanding the mechanism of flavor change and regulating the flavor quality in the Maillard reaction process, providing a new theoretical basis in the processing and development of *Oudemansiella raphanipes* products.

## 2. Materials and Methods

### 2.1. Materials and Reagents

*Oudemansiella raphanipes* was purchased from Shandong Century Intelligent Agriculture Technology. Xylose, glucose, fructose, galactose, and arabinose were purchased from Wanbang Industrial Co., Ltd. (Henan, China). L-cysteine (Cys), amino acid, and bromelain were purchased from Solabor Technology Co., Ltd. (Beijing, China). All other reagents were of analytical grade.

### 2.2. Preparation of EH

An appropriate amount of dry *Oudemansiella raphanipes* powder was added into deionized water to obtain a final substrate concentration of 9% (*w*/*v*). Then 300 U/mg bromelain (1.2%, *w*/*w*) was added and hydrolyzed at 55 °C for 3.5 h. After the enzymatic hydrolysis, the solution was heated at 100 °C for 15 min to inactivate the enzyme, and then centrifuged at 4000 r/min for 15 min. The supernatant was collected and taken for further analysis.

### 2.3. Preparation of MRIs and MRPs

EH and reducing sugar were used as raw materials and mixed uniformly at a certain mass ratio; the pH was adjusted to 7.5 using 3 mol/L NaOH. The samples (100 mL) were heated at a certain low temperature (50–90 °C) for a certain period of time; the solutions were immediately cooled with ice water to terminate the Maillard reaction. The samples were designated as MRIs. Afterwards, the samples (100 mL) were transferred to screw-sealed tubes and then placed in an oil bath maintained for 120 min at 120 °C, and cooled to room temperature before proceeding to the analysis and freeze-drying step with a vacuum freeze dryer (SCIENTZ-10, Ningbo Xinzhi Co., Ltd., Ningbo, China); the samples were designated as MRPs.

### 2.4. Single-Factor Experiment Design of MRI Reaction Conditions

The effects of different reaction conditions on the browning index were investigated, including reaction times (20, 40, 60, 80, 100 min), reaction temperatures (50, 60, 70, 80, 90 °C), reducing sugar species (arabinose, fructose, galactose, xylose, glucose), and the addition amount of reducing sugar (3%, 4%, 5%, 6%, 7%). The Cys tracing method was applied to measure the browning index of the Maillard reaction products, which was reflected by measuring the absorbance at 420 nm (A_420_) according to a UV-6100 UV–VIS spectrophotometer (Shanghai Yuanxi Instrument Co., Ltd., Shanghai, China) [16].

### 2.5. The Optimization by RSM

Studies have shown that MRIs can significantly improve the overall flavor characteristics of food protease hydrolysates, such as enhancing the perception of freshness or saltiness. It is found that the sensory evaluation score is directly proportional to the content of MRIs [16,17]. In addition, some studies have determined that the browning index of the final products after the second reaction stage (120 °C, 110 min) can be used as an indicator to characterize MRIs’ content, and the higher the concentrations of the MRIs, the lower the final values of the browning index, which is inversely proportional to MRIs’ content [3,4]. Therefore, the total score was calculated by the browning index and sensory score to investigate the response surface test.

$$\mathrm{Total}\;\mathrm{score}=\frac{\;(1\;-\;\mathrm{browning}\;\mathrm{index})\;*50\;*0.4+\mathrm{sensory}\;\mathrm{score}\;*50\;*0.6}{100}$$

Sensory analysis was conducted using the descriptive analysis method according to some researchers with some modifications [16,18]. Fifteen healthy panelists (10 females and 5 males, 20–28 years of age) were recruited from the Institute of Agro-Food Sciences and Technology, Shandong Academy of Agricultural Sciences (Jinan, China). Before the sensory test, these fifteen members needed to undergo a week of systematic sensory evaluation training according to the GB/T 16291.1-2012 standard [18]. Since there is a light aroma in MRIs, the taste exerts a significant effect on improving the taste of enzymatic hydrolysates [19]. According to the previous studies [3,20], the reference solutions were prepared by umami taste (0.8% sodium glutamate), sour taste (0.08% citric acid), sweet taste (5% sucrose), and bitter taste (0.005% quinine sulfate). All sensory tests took place in an air-conditioned room (22 °C) with isolated booths. The judges scored the magnitude of each taste from 1 to 7, where 1 was ‘‘low’’ and 7 was ‘‘high’’. The intensities of standard reference solutions were 4. Filtered water and white bread served as palate cleansers between samples. All scorecards were collected at the end of each session.

We adhered to the ethical principles of sensory research at the Agro-Food Sciences and Technology, Shandong Academy of Agricultural Sciences. These principles were reviewed by the Research Ethics Committee at the Institute of Agro-Food Sciences and Technology, Shandong Academy of Agricultural Sciences (Statement 19/2022). Written informed consent was obtained from each participant prior to participating in this study.

Based on the above single-factor experiment, the first stage reaction time (A: 55–65 min), reaction temperature (B: 55–65 °C), and amount of fructose (C: 4–6%) were taken as the independent variables, and the total score was taken as the response value; the response surface analysis based on the Box–Behnken design (BBD) was carried out to optimize the optimal conditions of preparing MRIs (Table 1).

### 2.6. Determination the Flavor Substances of EH, MRIs, and MRPs

#### 2.6.1. E-Nose

A commercial PEN 3 E-Nose (Airsense Analytics, GmBH, Schwerin, Germany) equipped with 10 metal oxide semiconductors (W1C, W5S, W3C, W6S, W5C, W1S, W1W, W2S, W2W, and W3S) was used to test the volatile aroma compounds of EH, MRIs, and MRPs. W1C is sensitive to aromatic compounds; W5S is sensitive to nitrogen oxides; W3C is sensitive to ammonia and aromatic compounds; W6S is mainly sensitive to hydrogen; W5C is sensitive to alkenes and aromatic compounds; W1S is sensitive to methane; W1W is sensitive to sulfide compounds; W2S is sensitive to alcohols, partially aromatic compounds; W2W is sensitive to aromatic compounds and organic sulfides; and W3S is mainly sensitive to alkenes. After pretreatment, the samples (10 mL) were placed in vials, capped with a PTFE–silicon stopper, and analyzed according to the study of Chen et al. [21]. First, the samples (EH, MRIs, and MRPs) in the bottle were kept at 60 °C for 10 min, and then the injection needle carrying the sample was inserted into the headspace bottle, and it was measured with the following conditions: sampling time of 1 s/group, sensor self-cleaning time of 80 s, sensor zeroing time of 5 s, sample preparation time of 5 s, injection flow of 400 mL/min, and analysis time of 80 s. Each sample was measured thrice.

#### 2.6.2. GC-IMS

The volatile compounds were analyzed using a FlavourSpec^®^ chromatography ion mobility spectrometry (GC-IMS) instrument (Gesellschaft für Analytische Sensorsysteme mbH (G.A.S.), Dortmund, Germany) equipped with a capillary column (MXT-5, 30 m × 0.53 mm × 1.0 μm) and an automatic headspace sampling unit (CTC-PAL, CTC Analytics AG, Zwingen, Switzerland). An amount of 2 g of the EH/MRI/MRP samples were placed in a 20 mL headspace vial and incubated at 60 °C for 15 min. The sample was driven by N_2_ into the chromatographic column at a temperature of 60 °C. IMS conditions were operated at a temperature of 45 °C with a drift gas flow rate of 150 mL/min.

#### 2.6.3. E-Tongue

The SA-402B E-Tongue (Insert, Tokyo, Japan) is used to measure the taste profile, which is composed of a Ag/AgCl electrode and an electrochemical sensor. An amount of 100 mL of the samples were poured into the special cup of E-Tongue at room temperature. Each sample was tested in four replicates, and the last three tests were used for the analysis. The E-Tongue device was self-tested and calibrated prior to the experiment. The potential value was converted into a sensory score value by an INSENT taste analysis system (Beijing Yingsheng Hengtai Technology Co., Ltd., Beijing, China).

#### 2.6.4. Sensory Evaluation of EH, MRIs, and MRPs

The method of sensory evaluation is the same as in Section 2.5.

### 2.7. Statistical Analysis

Each sample was measured three times. The results were shown as a mean ± standard deviation (SD) and were analyzed by SPSS 24.0 (SPSS Inc., Chicago, IL, USA) with one-way analysis of variance (ANOVA) and Tukey’s test to investigate the differences.

## 3. Results and Discussion

### 3.1. Single-Factor Analysis

Based on the mechanism of Cys indication on the MRI formation, the method for preparing the MRIs under low temperature in aqueous medium has recently been developed [14]. Referring to the evaluation method of Zhou et al. [16], Cys was added to the reaction system, reacting for different times within 50–90 °C, and then heated at 120 °C for 110 min. The interaction of Cys and MRIs inhibited the color formation [17]. Therefore, a change in A_420_ after the second high-temperature reaction stage could reveal the difference of MRIs’ concentration during the first low-temperature step. The higher the concentrations of MRIs, the lower the final values of A_420_.

**Figure 1:**
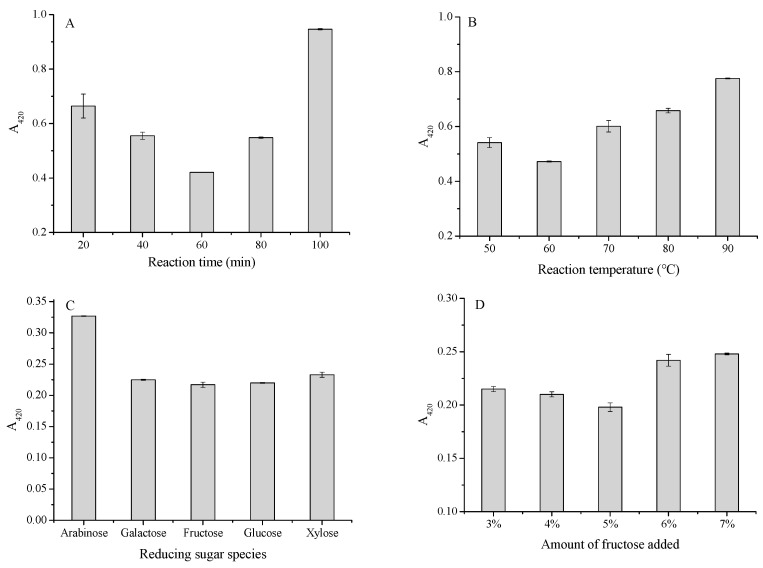
Effect of different reaction times (**A**), temperatures (**B**), reducing sugar species (**C**), and the amount of fructose (**D**) on the browning index of Maillard reaction. Note: the error bars are standard deviations (SD).

According to a previous study, the quality characteristics of conjugates were highly dependent on the Maillard reaction conditions, where the reaction time, reaction temperature, and types of reducing sugars were included [22]. It could be seen from Figure 1A that as the reaction time increased from 20 to 60 min, the A_420_ of the Maillard reaction system showed a downward trend. During the heating process, the melanoidins could be generated by Maillard reaction, the concentration of which increased with the extension of the reaction time. What is more, an inhibitory effect occurred due to the interaction between Cys and Maillard rearrangement products. Consequently, as the time prolonged, the higher the concentration of MRIs, the greater the interaction between MRIs and Cys, and the lighter the color [23]. At 60 min, it may be the critical point for the formation of MRIs. After 60 min, MRI would undergo enolization, deamination, dehydration, and fragmentation, as well as a series of sugar dehydration, and then the fragmentation products were generated, which could react with Cys to produce melanoidins, thereby increasing the browning index. Therefore, 60 min was chosen as the appropriate time in the MRI preparation.

As shown in Figure 1B, the browning index illustrated an increasing trend as the temperature increased from 60 to 90 °C, which might be attributed to the highest concentration of MRIs at 60 °C, and with the increase in temperature, it promoted the dehydration polymerization of reducing ketones and heterocyclic compounds and the reaction between Cys and fragmentation products to form melanoidins, leading to a higher browning index [24]. Here, 60 °C was determined as the optimal reaction temperature.

It is widely acknowledged that reducing sugars are one of the essences in the Maillard reaction [25], and the type of reactant was the main factor affecting the reaction rate based on the study of hydrolysate/peptide–polysaccharide Maillard reaction conjugates [26]. Figure 1C illustrates the lowest A_420_ when the fructose was taken as the reducing sugars, indicating that fructose could completely react with amino compounds with higher reactivity, thereby generating the highest concentration of rearrangement products. Cui et al. [15] found that the formation rate of an Amadori product from fructose–glycine in a glucose–glycine–sulfite solution increased rapidly with increasing time. Therefore, fructose was selected as the source of reducing sugars.

Figure 1D shows that the A_420_ of the second-stage Maillard product reached the minimum when the amount of fructose was 5%. As the addition of fructose gradually increased from 3% to 5%, the reaction between carbonyl compounds and amino compounds became increasingly obvious. When the amount of fructose was more than 5%, A_420_ showed an increasing trend, which might be ascribed to the excessive sugar, resulting in the overreaction of the Maillard reaction. After a successive process of dehydration and breakage, the fragmentation product of the excess sugar would react with Cys to produce overmuch melanoid [14]. Therefore, 5% was determined as the optimal fructose addition in aiming to obtain the maximum production of MRIs.

### 3.2. Optimization of Maillard Reaction Conditions by RSM

#### 3.2.1. Model Fitting and Statistical Analysis

The experimental design’s Maillard reaction condition and results (the yield of the total score) are shown in Table 1. The experimental results were analyzed by multiple regression analysis, and the second multinomial regression equation was obtained and expressed as follows:

Y = 37.7 + 0.40A − 0.37B − 0.70C − 0.30AB − 0.70AC + 0.10BC − 3.53A^2^ − 3.33B^2^ − 1.37C^2^ (1)

where A, B, and C are reaction time, temperature, and fructose amount, respectively.

Statistical analysis of variance (ANOVA) was applied to evaluate the reliability and suitability of the model, and the results are listed in Table 2. The model *F*-value was 20.93, and the *p*-value (0.0003) was smaller than 0.05, implying that the regression model was significant. The “Lack of Fit” (*F* = 2.57, *p* = 0.1919 > 0.05) was insignificant, indicating that the model established in this experiment has a good fit to the experimental results. The determination coefficient (R^2^ = 0.9642), the adjusted determination coefficient (R^2^adj = 0.9181), and the low coefficient variation (C.V. = 2.42%) further illustrated the excellent precision and fitness between the experimental values and the predicted values [27]. The Adeq Precision value was 11.674 (>4), manifesting the good reproducibility of the results. In Addition, Table 1 demonstrates that the linear coefficients (C) and quadratic term coefficients (A^2^, B^2^, and C^2^) significantly influenced the browning index and sensory evaluation of the MRIs (*p* < 0.05).

#### 3.2.2. Model Fitting and Statistical Analysis

The three-dimensional (3D) response surface and two-dimensional (2D) contour plots were built to explain the effect of the experimental factors on the total score of MRIs and their mutual interactions between the variables.

**Figure 2:**
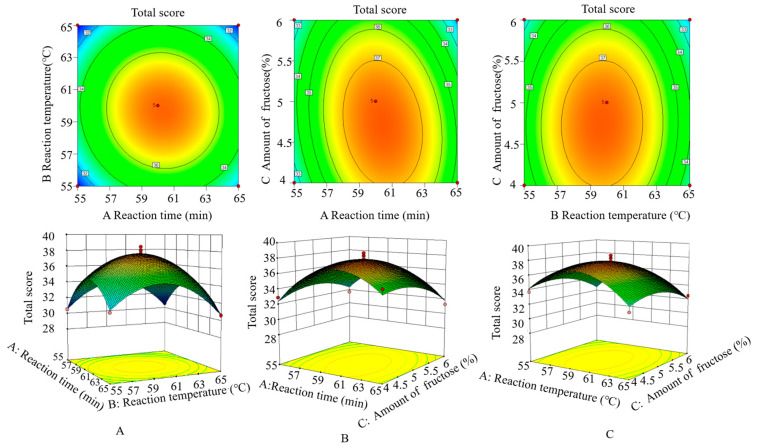
Response surface plots and contour plots displaying the effect of factor interactions on total score: (**A**) reaction time × reaction temperature, (**B**) reaction temperature × amount of fructose, (**C**) reaction time × amount of fructose.

Figure 2A shows the effect of reaction time and temperature on the total score of MRIs. It could be seen that when the amount of fructose was fixed at 5%, the total score first increased and then decreased with the increase in time and temperature, and reached the maximum when the time and temperature were 60.44 min and 59.68 °C. Moreover, it could be found from the elliptical contour plots that the interaction between reaction time and temperature produced an obvious effect on the total score. Similarly, as shown in Figure 2B,C, the interactions between the amount of fructose and time/temperature illustrated a significant difference.

Considering the actual operation error, the optimized process parameters were set as a temperature of 60 °C, a reaction time of 60 min, and a fructose addition of 5%, and the total score of the reaction product was 37.95 ± 0.09, which was basically consistent with the predicted value (37.83), confirming the accuracy and practicality of the equation.

### 3.3. E-Nose Detection and Analysis

**Figure 3:**
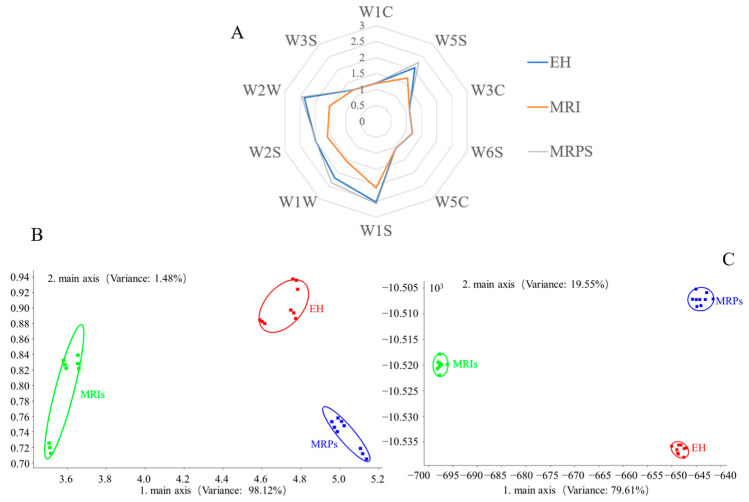
E-Nose radar profiles (**A**), PCA of the result of E-Nose (**B**), and LDA plots of reaction solution by E-Nose (**C**).

E-Nose is an intelligent sensory technology capable of discriminating different samples and providing a comprehensive flavor profile. In this study, an E-Nose equipped with 10 sensors was used to analyze the integrated flavor profile of the EH, MRIs and MRPs. As shown in Figure 3A, significant differences were observed in the signal intensity between EH, MRIs, and MRPs. The response intensity of the sensors W2W, W2S, W1W, W1S, and W5S was relatively high in EH and MRPs but low in MRIs, indicating that the contents of the aromatic compounds, sulfur compounds, alcohols, aldehydes, ketones, methyl components, and nitrogen compounds in MRIs were lower than those in EH and MRPs. As Maillard reaction had a significant effect on alcohols, aldehydes, ketones, sulfur compounds, hydrogen, nitrogen oxides, and methyl components, MPRs showed a higher response intensity than EH. According to principal components analysis (PCA), it could be seen from Figure 3B,C that MRPs significantly contributed to PC1 and PC2, showing that the complete Maillard reaction could increase the contribution rate of principal components and produce a more volatile aroma. However, MRIs with the least volatile aroma exhibited obvious differences in aroma compared with the MRPs; the results were in great agreement with previous research [14,28], which presented that Maillard reaction intermediates were important flavor precursors in the possession of stable physicochemical properties while with a weak aroma profile.

Moreover, there were some overlapping areas of the aroma profiles between MRIs and MRPs under PC1 projection, meaning a similar aroma distribution. Compared with PCA, linear discriminant analysis (LDA) distinguished the volatile flavor components of MRPs and MRIs. After two rounds of analyses, it could be concluded that the flavor differences between MRPs and MRIs were obvious, which was consistent with the results of E-Nose radar analysis.

### 3.4. GC-IMS Analysis

**Figure 4:**
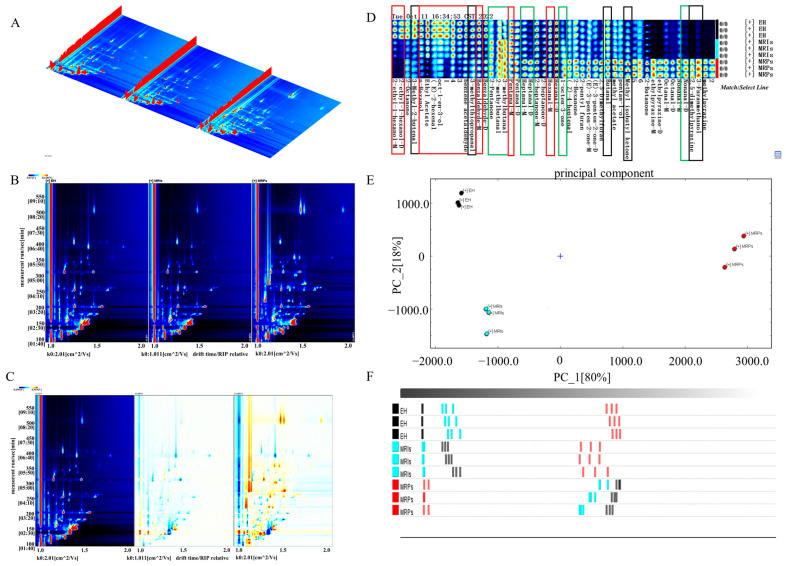
Three-dimensional spectrum of volatile substance composition (**A**), 2D spectrum of volatile substance composition (**B**), 2D differential spectrum of volatile substance composition (left: EH, middle: MRPs, right: MRIs) (**C**), GC-IMS fingerprint (**D**), PCA analysis (**E**), and Euclidean distance diagram based on GC-IMS (**F**).

GC-IMS is an innovative technology known for its rapid detection and simple pretreatment, offering a specific flavor profile that complements the capabilities of E-Nose. The volatile flavor compounds were qualitatively analyzed according to the NIST database and IMS database built-in GC-IMS. Volatile components by GC-IMS are shown in Table 3. In the 3D spectrum (Figure 4A) and the top view of the 2D spectrum (Figure 4B,C), clear visual distinctions can be observed among different samples (EH, MRPs, and MRIs) across a wide range of the GC-IMS spectrum.

From GC-IMS, a total of 35 volatile substances were detected in the three samples, including 6 alcohols, 13 aldehydes, 9 ketones, 2 esters, and 5 other compounds, whereas the volatile substance content of each sample was different. Combined with fingerprint analysis, phenylacetaldehyde, n-octanal, n-hexanol, 2-hexanal, 2-heptane, 2-methylbutyraldehyde, and 3-methylbutyraldehyde contributed to the fruity flavor in MRIs; nonanaldehyde, ethyl acetate, and methyl acetate provided the sweet aroma; and nonanaldehyde, 2-octanone, and CIS-4-heptenol constituted the milk and fat aroma. The color in Figure 4B,C represents the concentration of the substance, and the white means a low concentration, while the red means a high concentration.

Figure 4D shows the fingerprint of volatile components, representing the signal peak of each volatile substance in different samples; the redder the color, the higher the concentration of the substance. It could be seen from the green frame in Figure 4D that the content of heptaldehyde and 2-methylbutyraldehyde in MRPs was higher than that of EH, providing a fruity and clear fragrance. In addition, the content of nonanal was increased to amplify the grease taste, which might be attributed to the hydrolysis of free fatty acids, especially from the n-6 and n-9 polyunsaturated fatty acids [29,30]. As can be seen from the red frame in Figure 4D, as the reaction progressed, the content of 1-octen-3-ol, benzaldehyde, and valeraldehyde in MRIs was decreased, among which 1-octen-3-ol was a secondary hydroperoxide of fatty acids with a unique mushroom aroma, whereas benzaldehyde and valeraldehyde provided an almond flavor [31]. In addition, the presence of phenylacetaldehyde, 2-ethylhexanol, n-hexanol, 2-hexanal, n-hexanal, 2-methylbutyral, ethyl acetate in MRPs also improved fruity, fragrant, sweet, and cocoa flavors. The black frame in Figure 4D indicated that the content of 3-methylthiopropionaldehyde, 3-methyl-2-butenal, 2,5-dimethylpyrazine, 3-furanmethanol, 2-hexanone, n-butyraldehyde, 2-butanone, and 4-methyl-2-pentanone in MRPs was greatly higher than that of MRIs. Generally, 3-(methylthio) propionaldehyde with a pungent stench might result from the reaction of sulfur-containing amino acids (such as Cys, cystine, and methionine) and reducing sugars (such as ribose, glucose, xylose, and maltose) [32,33,34]. Other substances were more likely to be the Maillard overreaction products, such as melanogenoids and glycosylated end products, which were characterized with a pungent smell, bitter taste, and acetone smell, reducing the sensory quality of the entire Maillard reaction product remarkably.

PCA is a statistical analysis method that can simplify the original data by reducing dimensionality to change multiple variables into few variables [35]. As depicted in Figure 4E, the cumulative contribution rate of the first two principal components after dimensionality reduction was 98%. After feature compression, relatively complete information was still retained, which could better characterize the feature differences of the three samples. There was an obvious difference in the distribution of EH, MRIs, and MRPs on the PC_2 axis. EH and MRIs were close on the PC_1 axis, and their projection distances were far from MRPs on the PC_1 axis, indicating that the flavors of the three samples were quite different. As illustrated in Figure 4F, a Euclidean distance diagram based on GC-IMS showed that the distance between EH and MRIs was comparatively close, while EH and MRPs had the longest distance, and the distance between samples was much greater than the distance between parallel samples. This result was consistent with the PCA.

In summary, the preparation of MRIs could not only enhance umami on the basis of the original flavor of EH, but also prevent excessive Maillard reaction from producing MRPs with bad odors such as melanin.

### 3.5. E-Tongue Analysis and Sensory Evaluation of EH, MRPs, and MRIs

**Figure 5:**
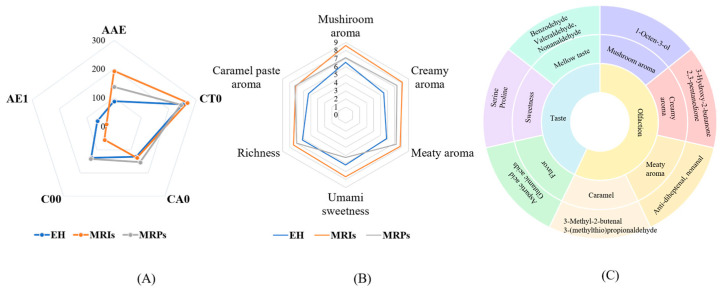
E-Tongue analysis (**A**), sensory evaluation results of three samples (**B**), and sensory wheel of MRIs (**C**).

E-Tongue and sensory evaluation were performed on EH, MRPs, and MRIs, and the results are shown in Figure 5. Among the five taste sensors in E-Tongue, AAE, CT0, CA0, AE1, and C00 sensors are used to test umami, salty, sour, astringent, and bitter tastes, respectively. The differences among the three reaction solutions can be clearly seen from Figure 5A. The Maillard reaction could improve the umami flavor of EH to a certain extent, and it was evident that the flavor-enhancing ability of MRIs was more prominent. In addition, the bitterness of MRIs was lighter than that of EH and MRPs; the result was similar to that in a previous report [16]. This indicated that MRIs could prevent the production of late glycation end products and inferior flavor substances such as melanoid, and also effectively remove the bad flavor of the enzymatic hydrolysate without producing an additional inferior flavor; all of the results were consistent with the fingerprint detection analysis.

Figure 5B shows the sensory characteristics of EH, MRPs and MRIs. It could be found that MRIs had higher scores of mushroom aroma, milk aroma, umami, and sweetness. After the Maillard reaction, all scores were increased, and the scores of MRPs were even lower than those of MRIs, suggesting that the Maillard reaction enhanced the overall flavor of EH, while the melanoid generated by the complete reaction was more likely to exert a negative effect on the flavor. On the whole, MRIs were the most excellent product to magnify the flavor of *Oudemansiella raphanipes*. In order to further describe the characteristic flavor of MRIs, a sensory wheel was constructed according to the method of Yu et al. [36] (Figure 5C). This flavor wheel was divided into two categories: smell (mushroom aroma, milk aroma, meat aroma, and caramel aroma) and taste (sweetness, umami, and richness). Furthermore, it was confirmed that as the Maillard reaction proceeded, all flavor characteristics were improved.

## 4. Conclusions

In this study, the two-stage Maillard reaction system of the *Oudemansiella raphanipes* enzyme hydrolysate was constructed, and the optimal conditions for the preparation of MRIs were obtained: fructose addition of 5%, reaction temperature of 60 °C, and reaction time of 60 min. The E-Tongue, E-Nose, and GC-IMS techniques and sensory evaluation were used to analyze the non-volatile and volatile flavor substances of EH, MRPs, and MRIs. In terms of non-volatile flavor substances, it was found that MRIs retained the unique mushroom aroma, enhancing the overall umami and sweetness, reducing the bitterness and astringency, and making the overall taste the most palatable. With regard to the volatile flavor substances, MRIs not only increased the content of umami and sweetness flavor substances, but also avoided the production of complete reaction products such as melanoid with inferior flavor, indicating that the preparation of MRIs of EH greatly exaggerated the original mushroom flavor, and further enhanced the umami taste of EH. The results will lay the foundation for the product development of *Oudemansiella raphanipes* and provide a theoretical basis for quality control and flavor changes during the processing of *Oudemansiella raphanipes*.

## Figures and Tables

**Table 1:** Maillard reaction condition optimization experimental design and results.

Test Number	A Reaction Time (min)	B Reaction Temperature (°C)	C Amount of Fructose (%)	Total Score
1	55	60	6	31.7
2	55	55	5	30.5
3	55	60	4	32.9
4	55	65	5	30.6
5	60	55	6	33.3
6	60	60	5	38.5
7	60	60	5	38.1
8	60	60	5	37.3
9	60	55	4	33.7
10	60	60	5	37.7
11	60	60	5	36.9
12	60	65	4	32.5
13	60	65	6	32.5
14	65	65	5	30.6
15	65	55	5	31.7
16	65	60	6	31.3
17	65	60	4	35.3

**Table 2:** Analysis of variance for regression models.

Source of Variance	Sum of Squares	df	Mean Square	*F*-Value	*p*-Value	Significance
Model	126.09	9	14.01	20.93	0.0003	*
A	1.28	1	1.28	1.91	0.2092	
B	1.12	1	1.12	1.68	0.2359	
C	3.92	1	3.92	5.86	0.0461	*
AB	0.36	1	0.36	0.54	0.4871	
AC	1.96	1	1.96	2.93	0.1308	
BC	0.040	1	0.040	0.060	0.8139	
A^2^	52.32	1	52.32	78.17	<0.0001	**
B^2^	46.55	1	46.55	69.55	<0.0001	**
C^2^	7.96	1	7.96	11.89	0.0107	*
Residuals	4.68	7	0.67			
Lack of Fit	3.08	3	1.03	2.57	0.1919	
Pure Error	1.60	4	0.40			
Cor Total	130.78	16				

Note: *, significant difference (*p* < 0.05); **, very significant difference (*p* < 0.01).

**Table 3:** Volatile components analysis by GC-IMS.

	Name	CAS#	Rt (sec)	Molecular Formula	Odor Description
Alcohols	Oct-1-en-3-ol	C3391864	330.108	C_8_H_16_O	Mushroom smell
	N-Hexanol	C111273	243.806	C_6_H_14_O	Fruity aroma
	(Z)-4-heptenal	C6728310	261.87	C_7_H_12_O	Grassy, oily, and creamy aroma
	3-Furanmethanol	C4412913	222.134	C_5_H_6_O_2_	Special bitter and spicy smell
	Pentan-1-ol	C71410	190.255	C_5_H_12_O	Special odor
	2-Ethyl-1-hexanol-M	C104767	393.983	C_8_H_18_O	Special odor
	2-Ethyl-1-hexanol-D	C104767	393.375	C_8_H_18_O	Special odor
Aldehydes	Nonanal-M	C124196	504.637	C_9_H_18_O	Sweet orange flavor, grease flavor
	Benzene acetaldehyde	C122781	406.768	C_8_H_8_O	Flowers, fruits
	Octanal-M	C124130	357.136	C_8_H_16_O	Intense fruity aroma
	Benzaldehyde-M	C100527	312.346	C_7_H_6_O	Almond flavor
	Benzaldehyde-D	C100527	311.188		
	Heptanal-M	C111717	263.059	C_7_H_14_O	Fruity, delicate
	3-Methylthiopropanal	C3268493	268.288	C_4_H_8_OS	Foul smell, strong onion smell
	(E)-2-Hexenal	C6728263	231.684	C_6_H_10_O	Leaf aroma, vegetable, and fruit aroma
	Hexanal-M	C66251	204.113	C_6_H_12_O	Grassy, winey
	Hexanal-D	C66251	203.4		
	Pentanal-M	C110623	164.536	C_5_H_10_O	Almond flavor
	2-Methylbutanal	C96173	154.399	C_5_H_10_O	Cocoa aroma, fruity aroma
	3-Methylbutanal	C590863	148.392	C_5_H_10_O	Fruity aroma
	Butanal	C123728	124.551	C_4_H_8_O	A suffocating pungent odor
	3-Methyl-2-butenal	C107868	210.292	C_5_H_8_O	Pungent smell
Ketones	2-Octanone	C111137	344.587	C_8_H_16_O	Smell of milk, cheese, mushrooms
	2-Heptanone-M	C110430	257.117	C_7_H_14_O	Fruity aroma
	2-Heptanone-D	C110430	255.691		
	1-Octen-3-one	C4312996	330.562	C_8_H_14_O	—
	2-Hexanone	C591786	198.288	C_6_H_12_O	A pungent odor similar to acetone
	(E)-3-Penten-2-one-M	C3102338	181.431	C_5_H_8_O	—
	2-Pentanone	C107879	159.843	C_5_H_10_O	Acetone-like odor
	2-Butanone	C78933	133.186	C_4_H_8_O	Acetone smell
	(E)-3-Penten-2-one-D	C3102338	179.93	C_5_H_8_O	—
	Methyl isobutyl ketone	C108101	178.305	C_6_H_12_O	Strong aldehyde odor
Esters	Methyl acetate	C79209	119.483	C_3_H_6_O_2_	Honey aroma
	Ethyl acetate	C141786	137.504	C_4_H_8_O_2_	Fruity, sweet
Other compounds	2-Pentyl furan	C3777693	343.538	C_9_H_14_O	Fruity and vegetable, earthy flavor
	2,5-Dimethylpyrazine	C123320	277.372	C_6_H_8_N_2_	Pungent aroma of fried flowers and chocolate, cream smell
	Ethylpyrazine-D	C13925003	276.006	C_6_H_8_N_2_	Nutty, roasted, meaty
	Ethylpyrazine-M	C13925003	276.663		
	Methylpyrazine	C109080	219.29	C_5_H_6_O_2_	Nutty, musty, toasted
	2,5-Dimethylfuran	C625865	167.728	C_6_H_8_O	—

Note: “—” means no description of this substance.

## Data Availability

The original contributions presented in the study are included in the article, further inquiries can be directed to the corresponding author.
